# A Systematic Review of Sensor Fusion Methods Using Peripheral Bio-Signals for Human Intention Decoding

**DOI:** 10.3390/s22176319

**Published:** 2022-08-23

**Authors:** Anany Dwivedi, Helen Groll, Philipp Beckerle

**Affiliations:** 1 Chair of Autonomous Systems and Mechatronics, Department of Electrical Engineering, Friedrich-Alexander-Universität Erlangen-Nürnberg, 91052 Erlangen, Germany; 2 Department of Artificial Intelligence in Biomedical Engineering, Friedrich-Alexander-Universität Erlangen-Nürnberg, 91052 Erlangen, Germany

**Keywords:** myography, data fusion, human-intention decoding, muscle–machine interfaces

## Abstract

Humans learn about the environment by interacting with it. With an increasing use of computer and virtual applications as well as robotic and prosthetic devices, there is a need for intuitive interfaces that allow the user to have an embodied interaction with the devices they are controlling. Muscle–machine interfaces can provide an intuitive solution by decoding human intentions utilizing myoelectric activations. There are several different methods that can be utilized to develop MuMIs, such as electromyography, ultrasonography, mechanomyography, and near-infrared spectroscopy. In this paper, we analyze the advantages and disadvantages of different myography methods by reviewing myography fusion methods. In a systematic review following the PRISMA guidelines, we identify and analyze studies that employ the fusion of different sensors and myography techniques, while also considering interface wearability. We also explore the properties of different fusion techniques in decoding user intentions. The fusion of electromyography, ultrasonography, mechanomyography, and near-infrared spectroscopy as well as other sensing such as inertial measurement units and optical sensing methods has been of continuous interest over the last decade with the main focus decoding the user intention for the upper limb. From the systematic review, it can be concluded that the fusion of two or more myography methods leads to a better performance for the decoding of a user’s intention. Furthermore, promising sensor fusion techniques for different applications were also identified based on the existing literature.

## 1. Introduction

In the last decade, robotic and prosthetic devices are gaining importance in everyday tasks (e.g., robotic teleoperation and telemanipulation, user assistance, rehabilitation and augmentation). For an intuitive user experience while interacting with and controlling these devices, new human–machine interfaces need to be developed [1]. Current interfaces that facilitate such interactions often include joysticks and mechanical buttons. A drawback of these interfaces is that they require a steep learning curve for the user to map complex user motions to simple joystick motions or buttons. This results in an inefficient control of the robotic or prosthetic devices due to limited functionality offered by the interface. An alternative approach to interface with such devices is to employ muscle–machine interfaces (MuMIs) [2]. Such interfaces can be developed employing different sensing modalities to measure the muscle activity or movement. Some of the most common modalities are, but not limited to: electromyography (EMG) [3,4,5,6,7,8,9,10], ultrasonography (US) [11,12,13,14,15], mechanomyography (MMG) [16,17,18,19], and near-infrared spectroscopy (NIRS) [20,21].

EMG measures the electrical activation in the muscles which are generated as a result of biological processes during the contraction of the muscles [22]. These activations can be measured both invasively, using needle electrodes, and non-invasively, by placing electrodes on the surface of the skin. The non-invasive method, known as surface EMG (sEMG), is more common and sees a wide range of applications [23,24,25,26,27,28]. Generally, EMG and sEMG are used to denote the non-invasive surface EMG method. Being non-invasive, these systems are easy to use. EMGs also have a high temporal resolution [29]. However, EMG-based methods are non-stationary, prone to crosstalk between different muscles, sensitive to electrode shifts during their use, sweating and fatigue, and electro-magnetic noise [30]. Moreover, they can only be used to measure surface muscles, as to measure the activations of the deep-seated muscles, the invasive variant is needed [31].

An alternative to EMG is MMG. MMG measures the mechanical response of the muscles during contraction [32]. For MMG, the information is usually in the frequency band of 2–200 Hz. In the literature, it is referred to as several different terms, such as: muscular sound, phonomyogram, acoustic myogram, soundmyogram or vibromyogram [33]. Some of its advantages over EMG are that it is not affected by sweat, has a higher signal-to-noise ratio, and is less sensitive to the variations in placement of the sensor on the muscle of interest [34,35]. However, a few drawbacks to this approach are that it is prone to crosstalk between different muscle groups muscle EMG [36]. Furthermore, interference due to ambient acoustic/vibrational noise as well as lack of established sensors inhibits its mainstream use [32].

Other alternative myography methods US and NIRS. US utilizes ultrasound images to photograph muscle movement, which are then used to decipher the user’s intention [37]. In the literature, US is also referred as sonomyography. An advantage of US over other myographies is that it can record the activity of deep muscles non-invasively without cross-talk from adjacent muscles [11]. It is also robust against sweat and electrical interference. However, US-based interfaces are generally bulky and expensive, and the US probe needs to be frequently gelled for proper functioning. NIRS decodes user intention by quantifying the relative changes in the concentration of oxygenated and deoxygenated hemoglobin during muscle contraction [20]. It offers high spatial resolution while tracking user motion, but it is sensitive to muscle fatigue and optical noise [38]. Moreover, NIRS has a delayed response to the motion of the user, leading to a low temporal resolution [39].

From the available literature, it is evident that the user intention can be decoded to execute various tasks using different sensing modalities. Researchers have employed MuMIs for decoding hand gestures to control robotic hands or interaction with computer application [40,41,42,43], decoding continuous arm–hand motions [44,45,46], rehabilitation after strokes [47,48], and for games and entertainment [49]. Such MuMIs have also been employed in decoding walking patterns for an effective control of lower limb prosthesis [50,51], quantifying user fatigue during various tasks [52], and for decoding user intentions during collaborative tasks with robots [53,54]. Several researchers have also focused on employing electroencephalography signals to decode user intentions, hand and finger motions, decoding walking intentions etc. [55,56,57,58,59]. Since the main focus of this work is on peripheral bio-signals, electroencephalography-based studies are not included in the systematic review process. While all available methods have their advantages and drawbacks, it appears that the performance of the individual methods can be improved by fusing different methods together or by performing fusion with other sensors (e.g., inertial measurement units (IMUs)). This work presents different methods of acquiring bio-signals from the peripheral nervous system of the user in a non-invasive way to decode the user’s intentions. The goal of this work was to analyze the advantages and disadvantages of different myography methods and to explore the properties of different fusion techniques in decoding the user’s intentions by reviewing and discussing different fusion methods. By finding the advantages and disadvantages of different sensing modalities, informed decisions can be taken to select sensing methods while developing MuMIs. To do this, we performed a systematic review of the works that employ the fusion of different sensors and myography techniques.

The rest of the paper is organized as follows: In Section 2, we present the details regarding the process followed for the systematic review. In Section 3, we present the results along with a description of the key papers included in the review. The papers that emphasize the potential of using fusing different myography methods and external sensors in the applications of decoding human intention are considered in Section 4 to give a broader overview. Finally, Section 5 presents the concluding remarks and potential future directions for the research.

## 2. Methods

The literature for the systematic review was searched during the month of September in 2021. The databases and the search terms are listed in Table 1. The term in the right column of Table 1 is exemplary of the syntax used, however, it was adjusted as per the requirements of the search engine of each database while making sure that they still are semantically identical. For a comprehensive study, a full-text search was conducted across all databases. Full-text searches included title, keywords, and research highlights along with the full-text papers, depending upon the database. For the whole process of the systematic review, the Preferred Reporting Items for Systematic Reviews and Meta-Analyses (PRISMA) guidelines were followed [60,61]. It provides instructions to perform a review using systematic methods to collate and synthesize findings of studies that address a clearly formulated question.

**Figure 1:**
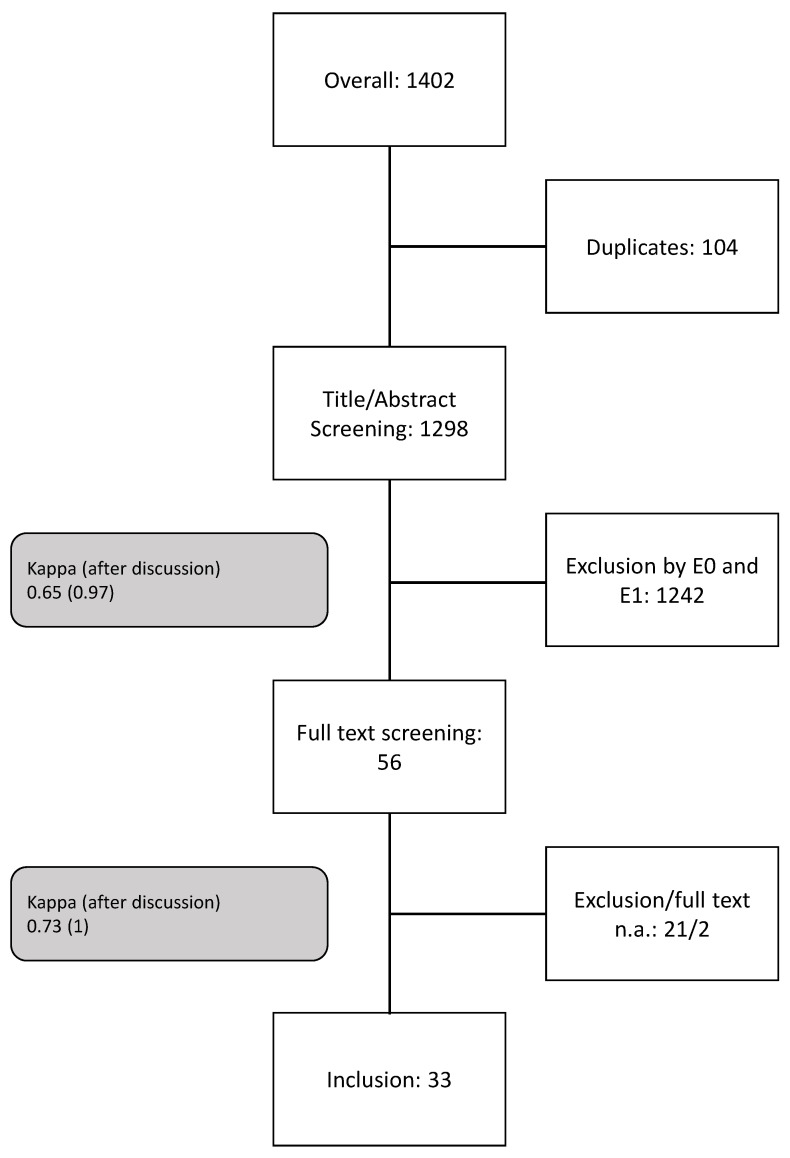
Overview of the screening process for selecting the studies for the systematic review. The grey boxes indicate the Cohen’s Kappa values before and after (in brackets) discussion.

Three exclusion (E) and one inclusion (I) criteria were chosen by all authors to determine the relevance of the papers. The screening and assessment process of the papers is shown in Figure 1. The search through the databases yielded 1402 papers which, after removing the duplicates, were reduced to 1298 for the first screening. In the first round of screening, only titles and abstracts were considered which were diligently excluded considering exclusion criteria E0 and E1. During the second screening round, the full texts were read, with an additional consideration of the inclusion criterion I0 and exclusion criterion E2. Studies were included only if they satisfied criterion I0, but excluded as soon as any of the exclusion principles were met. The exclusion and inclusion criteria considered in the study are as follows:

- E0: Studies focusing on animals, the analysis of vital signs, humans under 18, electrical stimulation;
- E1: A fixed structure (should have the potential to be portable to be included), stationary instrumentation
- E2: No inclusion criteria met
- I0: Should use two or more sensing methods focusing on skeletal muscles and perform fusion to achieve better performance

Inclusion criterion I0 covers both, theoretical and experimental contributions, based on which the subsequent survey and the results are structured. E0 excludes all studies that are conducted with animals, as well as, all the studies that employ different myographies and data fusion techniques for studying vital signs to monitoring health conditions were removed. Furthermore, a lot of works focus on the development on muscle strength in children, as this is not the main focus of the current review, the studies with humans under 18 were excluded. We also excluded all studies with electrical stimulation as they focus on actuating the muscles to help users regain control of their muscles (as part of therapy and rehabilitation) after a stroke as opposed to decoding the intention of the user. E1 focuses on the wearability of the interface and excludes the works which employ fixed structures for the experimental rig, such as: magnetic resonance imaging, computed tomography, positron emission tomography, etc. Finally, E2 excludes the papers that do not meet the inclusion criteria. Strict inclusion/exclusion criteria were set up for the selection of the papers focusing on the fusion of different sensing technologies targeting user intention decoding, however, unselected papers that emphasize the potential of such applications are considered in the discussion to give a broader overview.

All papers were separately screened by the first and second author. To assess the inter-rater agreement, Cohen’s Kappa value was calculated [62]. Cohen’s Kappa value is an established measure in the research community and was suggested as a statistical measure of inter-rater reliability [63,64,65]. There are several different ways to interpret Cohen’s metric. One of the proposed methods is as follows (other interpretations can be found in Section 3 with the discussion of the results): values < 0 as indicating no agreement and 0.00–0.20 as “none”, 0.21–0.39 as “minimal”, 0.40–0.59 as “weak”, 0.60–0.79 as “moderate”, and 0.80–0.90 as “strong” and >0.90 as “almost perfect” agreement [66]. If the two raters ($R_{1},R_{2}$) mark their inclusion and exclusion responses as follows: $R_{1,yes}R_{2,yes}=a$, $R_{1,yes}R_{2,no}=b$, $R_{1,no}R_{2,yes}=c$, and $R_{1,no}R_{2,no}=d$. Then, the Cohen’s Kappa can be calculated as:

(1) $$\kappa =\frac{p_{o}-p_{e}}{1-p_{e}}$$

where $p_{o}$ is the relative observed agreement among raters, and $p_{e}$ is the hypothetical probability of chance agreement. $p_{o}$ and $p_{e}$ can be calculated as follows:

(2) $$p_{o}=\frac{a+d}{a+b+c+d}$$

(3) $$p_{yes}=\frac{a+b}{a+b+c+d}\times \frac{a+c}{a+b+c+d}$$

(4) $$p_{no}=\frac{c+d}{a+b+c+d}\times \frac{b+d}{a+b+c+d}$$

(5) $$p_{e}=p_{yes}+p_{no}$$

The papers with conflicting ratings were discussed and the decisions were revised and were included only if it was included by both authors. By following this approach, premature exclusion or inclusion can be avoided. The screening process was designed and supervised in collaboration with the third author.

## 3. Results

An overview of the screening process is shown in Figure 1 and discussed in detail in Section 2. From the 1298 papers that were reviewed during the first round of screening, 1199 papers were excluded by both the authors and 49 papers were excluded by only one author based on reading the title and abstract before a discussion between them. The remaining 50 papers were included by both authors. After resolving conflicting ratings in a discussion, 1239 papers were excluded by both the authors and 3 papers were excluded by only one author. Finally, 56 papers were included for the second screening round. In the first round of screening before a discussion between the authors, a Cohen’s Kappa value of κ=0.65 was achieved, which suggests a “good” [67] or “substantial” [68] or a “moderate” [66] inter-rater agreement. However, after discussion between the authors, the Cohen’s Kappa value was increased to κ=0.97, suggesting an “almost perfect” [68] inter-rater agreement.

During the second round of screening, the papers were included or excluded based on a full-text analysis. In this round, an English manuscript (or an official translation) of the two papers was not found and due to a lack of expertise to translate the manuscripts by the authors the papers were excluded from the study. From the 56 papers that were reviewed during this round, 17 papers were excluded by both the authors before a discussion between them, while 7 papers were excluded by only one author. The remaining 30 papers were included by both the authors. However, after a discussion, a complete agreement was reached and 33 papers were included and 21 papers were excluded by both the authors. In the second round of screening before discussion between the authors, a Cohen’s Kappa value of κ=0.73 was achieved suggesting again, a “substantial” [68] or a “moderate” [66] inter-rater agreement. However, after a discussion between the authors, a total agreement was achieved making the Cohen’s Kappa κ=1.

All the individual myography methods have respective advantages and disadvantages: EMG-based systems, being non-invasive, are easy to use and also have a high temporal resolution. However, such methods are sensitive to electrode shifts during their use, sweating and fatigue, and electro-magnetic noise. MMG-based systems have a higher signal-to-noise ratio, and are less sensitive to the variations in the placement of the sensor on the muscle of interest; however, interference due to ambient acoustic/vibrational noise as well as the lack of established sensors inhibits its mainstream use. US can record activity of deep muscles non-invasively without cross-talk from adjacent muscles, but US-based methods are usually bulky and expensive. NIRS-based systems offer high spatial resolution while tracking user motion, but are sensitive to muscle fatigue and optical noise as well as have a delayed response to the motion of the user, leading to a low temporal resolution. Table 2 lists the papers included in the systematic review. In this table, we also show the different myography methods and external sensors used in the paper to study performance enhancement using data fusion. Table 2 also discusses the properties of different fusion methods as well. In the following subsections, we present the fusion different methodologies explored in the studies.

### 3.1. Fusion of EMG and MMG

Tkach and Hargrove [69] employed EMG and MMG data to allow ambulation over various types of terrains. Furthermore, they compared the performance of EMG, MMG and fused EMG and MMG, where they concluded that sensor fusion performs better than individual methods. In [70], the authors presented a custom sensor to acquire EMG and MMG activations simultaneously. In this study, the MMG data acquisition is implemented using optical sensors. This proposed method is less susceptible to motion artefacts, as compared to when implemented using accelerometers. An advantage of data fusion with MMG is that meaningful data can be acquired even during passive motions, during which EMG is non-observable. This is because the passive motion has involuntary changes in the physiological muscle cross-sectional area resulting in passive extension and flexion of the limb, and such passive motions are performed without any myoelectric activations. In [71], Tsuji et al. proposed a fusion of EMG and MMG to distinguish between patients that are susceptible to bilateral patellar tendon hyperreflexia and patients with no prior history of neurological disorders. They observed a significantly high amplitude for the root mean square and low values of mean power frequency for the rectus femoris, vastus medialis and vastus leteralis muscles. This observation is true for both EMG and MMG with both maximal and constant force. They conclude that employing both EMG and MMG for objectively quantifying the patellar tendon reflex is simple and desirable for future clinical applications.

### 3.2. Fusion of EMG and US

In [73], Botter et al. focused on analyzing the effects on the signal quality due to interference with different sensors during multi-modal data acquisition. The analysis is based on the muscles of the leg of the participant. It was concluded that the US data with and without EMG electrodes was comparable, but the electrode–skin impedance of the employed EMG-US electrodes was higher than that of conventional EMG electrodes. However, despite higher impedance values, the electrode–skin noise levels of the EMG-US electrode was comparable to the conventional EMGs. In [74], Yang et al. compared EMG and US separately and also the fusion of EMG and US while decoding eight different hand gestures and discrete and continuous estimation of forces being exerted. They concluded that a fusion-based sensor is better than individual sensing methodologies, since US performed better than EMGs while decoding discrete gestures and discrete force estimations. However, EMG-based decoding models performed better during continuous force estimation. The studies suggest that the addition of a US sensor with the EMG electrodes increase the skin impedance values. However, the fusion of US and EMG is desirable, especially in cases where a combination of discrete and continuous intention is decoded (e.g., the classification of different grasp types and decoding the respective grasping forces for picking up objects using a prosthetic hand).

### 3.3. Fusion of EMG and NIRS

Guo et al. [38,39,75] employed the fusion of EMG and NIRS for hand gesture classification in both human–computer interaction applications and the control of upper limb prosthesis. In their work, they concluded that the fusion of EMG and NIRS leads to a better decoding performance as opposed to developing interfaces independently with each myography method. While in [76], Paleari et al. employed the same combination of sensors in the classification of hand motion instead of hand gestures and reach the same conclusion—that the fusion data performs better.

### 3.4. Fusion of EMG and Accelerometers

Bio-signals are also fused with other sensing methods, such as accelerometers and IMUs. The fusion of EMG with accelerometers is one of the most explored fusion techniques. A reason for this could be the commercial availability of this combination with the Delsys EMG bioampliers [102] and the Myo armband by Thalmic labs [103]. Several studies have employed it in decoding human arm–hand gestures and motions. Fougner et al. [78] utilized EMG and accelerometer fusion for the classification of limb position in various orientations. They showed that decoding models developed using EMG data from multiple arm orientations performed better compared to when it is developed using data from only one arm orientation but tested for gesture execution in all the orientations. However, further improvement in the performance can be achieved by performing two-stage classifications, where, in the first stage, the accelerometer data are employed followed by an arm orientation-aware EMG-based gesture classification. In [80,81], Gijsberts et al. utilized the NinaPro database [104] to demonstrate the need for the accelerometer data in decoding the human hand gestures. They concluded that the highest accuracy is obtained when both modalities are integrated in a multi-modal classifier. Furthermore, they proposed a movement error rate as a new metric of evaluation, since a drawback of the window-based accuracy is that it does not distinguish between different types of mistakes made by the classifier (e.g., errors due to misclassifications and prediction delay). In [85], Wang et al. employed the NinaPro database to compare the decoding performance of support vector machines, convolutional neural networks, and recurrent convolutional neural networks. They concluded that recurrent convolutional neural networks along with fusion of EMG and accelerometer data perform better than other decoding methods. Wang et al. further confirmed this finding on a custom dataset collected which was collected during the study [85]. The fusion of EMG and accelerometers is also used for the classification of different types of tremors of Parkinson’s disease [79]. Here, the authors concluded that the combination of the two sensing methods leads to an increased performance of classifying tremors.

This sensor fusion is also employed in improving the performance of a lower limb prosthesis as well. In [83], Joshi and Hahn proposed a framework to perform seamless transitions between different terrains. They detected the direction (ascent or descent) and terrain (ramp or stairs) patterns when a person transitions from over ground to stairs or ramp locomotion. Furthermore, they showed that EMG and accelerometer data sources are complementary across the transitional gait cycle, and concluded that sensor fusion will lead to a robust classification. While in [84], Gupta et al. classified nine activities of daily living related to walking (sit, stand, lay down, walk on level: normal and high speed, walk up/down stairs, walk up/down ramp). They too reached a conclusion that the fusion of EMG and accelerometer data leads to an improved performance of the system.

### 3.5. Fusion of EMG and IMU

Wu et al. presented a framework based on the fusion of EMG and IMU data for decoding Chinese sign language and tested its performance and capabilities over two studies. In the first study [82], they decoded 40 different gestures and later extended it to decoding 80 gestures [87]. In this study, four different classification methods were compared (naive Bayes, nearest neighbor, decision tree, and support vector machines), with nearest neighbor and support vector machines performing better than the others. Furthermore, the performance of each classifier was compared for just IMU data and the fusion of EMG and IMU. It was observed that the performance improves with the fusion data. In [88], Yang et al. focused on one-handed and two-handed gestures recognition in Chinese sign language and show that the fusion of the EMG, accelerometer, and gyroscope data performs significantly better than if each method was used independently, or the EMG was combined with the accelerometer, the EMG was combined with the gyroscope and accelerometer was combined with the gyroscope.

In [89], Fang et al. decoded five gestures for a real-time control of a turtlebot. Different feature extraction methods were compared and it was concluded that the use of both time-domain and time-frequency domain leads to a better performance. On the contrary, in [91], the authors utilized a fusion of EMG and IMU data for a control of robot arm. In this work, EMG was used to tune the joint stiffness of the robot, while IMUs were employed to teleoperate the robot arm.

### 3.6. Fusion of EMG and Accelerometer with Optical Sensing

In [92], Yoshikawa et al. studied a fusion of EMG data with an optical distance sensor. They optical sensor measures the distance between the skin and the sensor, and it was hypothesized that the distance changes caused by muscle elevation can compensate the limited information derived from myoelectric activities (e.g., low myoelectric activations for arm pronation and supination). From the results, it can be seen that the hybrid approach leads to a better gesture classification performance, especially with pronation and supination tasks. In [93], Luan et al. implemented a fusion of optical sensors with accelerometer data for the classification of hand gestures. For the fusion of the two data streams, a dynamic time warping method was employed. It was noted that the performance of the hybrid system is better than just using the accelerometer for classification.

### 3.7. Fusion of MMG and IMU

In [94,95], Woodward et al. developed a fusion of MMG and IMU data. The MMG data acquisition was implemented using microphones. In [94], experiments were conducted in three sets. In the first set, MMG was compared with EMG for signal onset, held contraction and offset of muscle activity. In the second set of experiments, MMG and IMU data are combined to determine gait and muscle contraction changes following a restriction in knee and ankle flexion/extension. Finally, the third set investigates uncontrolled and prolonged data collection to demonstrate the pervasive nature of the technology while showing that it can be employed to determine active periods. In [95], they observed a significant (*p* < 0.01) progressive change in muscular activity in subjects with atypical gait over time. Furthermore, in their experiments with the walking patterns of the user, the inclusion of MMG data with IMUs significantly improved the accuracy of recognizing the activities when running is a more abundant activity.

In [96], an armband was developed, fusing MMG and IMU data, to recognize different hand gestures for the control of a quadrotor. Five gestures were decoded with an average accuracy of 94.38%, with the lowest gesture decoding accuracy of 85.64%. In [97], different symptoms of Parkinson’s disease were classified using MMG and IMU data. Furthermore, the decoding models were developed to distinguish between healthy subjects and Parkinson’s disease patients. It was found that the inclusion of MMG-based features significantly improves the decoding accuracy of rigidity-based PD symptoms. However, the removal of MMG-based features does not affect the classification accuracy for a kinetic tremor and rest tremor, but the accuracy decreases for bradykinesia and postural tremor.

### 3.8. Fusion of EMG, US and MMG

In [98], Chen et al. utilized the fusion of EMG, US, and MMG to study the behavior of the rectus femoris muscle during isometric contractions. They employed local polynomial regression to reveal nonlinear and transient relationships between multimodal muscle features and torque. The authors concluded that the proposed multimodal method can provide complete and novel information on muscle contraction. In [99], Han et al. introduced a new outlier detection method to facilitate the detection of transient patterns of muscle behavior during isometric contraction.

### 3.9. Fusion of EMG, MMG and NIRS

In [100], Ding et al. presented a method utilizing the fusion of EMG, MMG, and NIRS, to study the relationship between the different sensing technologies during incremental force execution. The authors noticed that the signal intensity increases with the increasing force for both EMG and MMG. However, for NIRS, the trend is not obvious between 50% and 80%. They attribute this observation to the increased pressure of muscles and blood vessels as a result of increasing forces being exerted, which leads to a limited flow of blood in the blood vessels [105]. In [101], the authors developed a fused sensor for simultaneous data acquisition with EMG, MMG, and NIRS as sensing modalities. They observed that the classification accuracies for the decoding models developed using all the three sensing modalities is better than when developed using just EMG or a combination of EMG and NIRS. Furthermore, the models developed using EMG and NIRS outperformed those developed only using EMG.

## 4. Discussion

With the increasing demand for intuitive interfacing methods for various computer applications, robotic systems, or prosthetic and rehabilitation devices, new ways need to be explored to acquire information from the user to facilitate intention decoding. The present survey aimed at exploring different sensor fusion methods employed using peripheral bio-signals to develop such intention-decoding frameworks. The included studies evaluated different fusion methods and reached the conclusion that the fusion of two or more sensing methods leads to an increased performance in terms of decoding human intention. However, from the current survey, it is inconclusive to say which is the best combination for this purpose.

**Figure 2:**
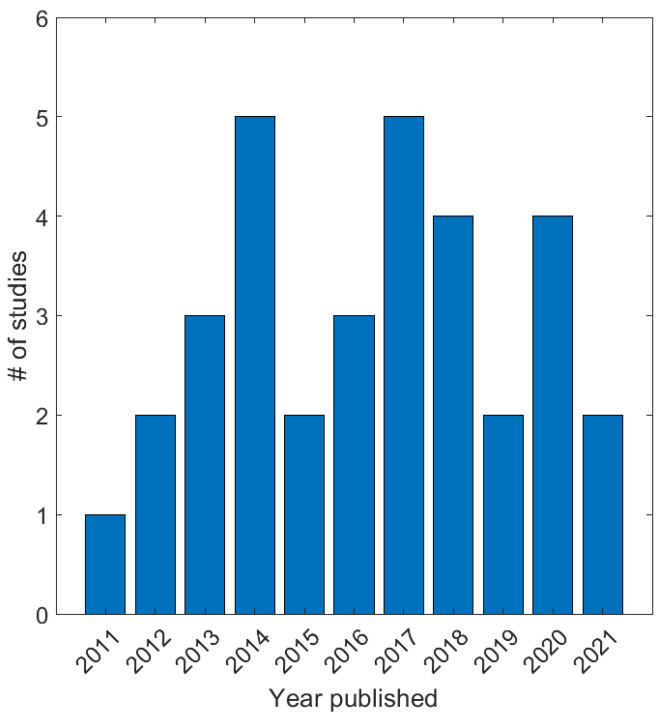
Distribution of the selected studies based on the year of publication.

Figure 2 shows the distribution of the studies that were selected for this review over the last decade. It can be noticed that the interest in the fusion studies has been continuous over this period of time. However, from Table 3, it can be noticed that the EMG-based sensing are the most common methods. This can be attributed to widely available commercial data acquisition systems that include both equipment for research and analysis [102,106,107] in the field as well as hobby kits [108]. The second most explored myography method that is explored for fusion study is that of MMG-based systems. These are followed by NIRS and US, respectively. The reason for the popularity of MMG over NIRS and US can be attributed to the hardware needs, as the MMG data can be acquired using accelerometers or microphones. A lot of studies also consider the fusion of myography methods with accelerometers or IMUs for intention decoding.

The majority of the studies have focused on upper limb intention decoding. More precisely, the main topic of interest was to decode the gestures executed by the users using their hands to either control a robotic device [91,96], sign language decoding [82,87] or for prosthetic applications [80,81]. For this, studies have focused on both the classification of hand gestures as well as the classification of hand motions [78,109]. Few studies have focused on the diagnosis of symptoms of Parkinson’s disease as well as on identifying healthy participants from the patients of Parkinson’s disease [79,97]. Studies have also focused on lower limbs. Such studies have generally aimed to classify walking patterns (e.g., walking on the level surface, climbing up/down stairs, etc.) of the user as well as the terrain that the user is walking on for a better support from the leg prosthesis to facilitate a better walking experience [70,71,84,94,95]. For decoding continuous motions, EMG or MMG with accelerometers or IMU appears to be more promising, while in tasks that require both the classification and regression of user intention, a fusion of EMG and US can be employed. In cases where the timing of the decoding has high preference, NIRS may not be the ideal choice due its low-time resolution. NIRS-based interfaces also have difficulties in decoding forces exerted by the users while performing various tasks [100], however, a combination of EMG, MMG, and NIRS may be used in recognizing the onset of muscular fatigue [101].

For user intention decoding, the most common muscle sites were the extensor digitorum muscle group, the flexor digitorum muscle group, and the muscles around the radio-humeral joint. A few studies have also employed the bicep and tricep muscles. However, studies that acquired the myoelectric activation from the bicep and tricep muscles were interested in the classification of the user’s gesture that was either executed with various arm orientations or required arm motions [78,80,81,97]. In the studies focusing on the lower limbs, the main muscles groups utilized for decoding user intention are rectus femoris, vastus medialis, and vastus leteralis.

Different sensing methods require different signal conditioning methods. For EMG signals, the most studies employed a bandpass filter of 20–500 Hz. The MMG data was generally bandpass filtered between 10 and 100 Hz. However, the sampling rate for both the myographies was usually 1000 Hz. For NIRS data, most studies employed a lowpass filter of 300 Hz and a data sampling rate of 1000 Hz. US data do not require a specific data filtering step and they were captured as images with a frame rate of 25 Hz. The accelerometer and the IMU data were also used in the raw form without the application of any data filtering techniques. Regarding feature extraction methods, the time domain features were extracted in the majority of the EMG and MMG-based studies with root mean square value, mean absolute value, zero crossings, and waveform length being some of the common ones. Time domain features are usually the most common ones as they are computationally less complex to calculate, and thus more suitable for real-time applications [110]. The second most common feature types were frequency domain features and finally the time-frequency features. The window size for the extraction of features was usually between 250 and 300 ms, while the window slide was between 50 and 100 ms. The window size should be carefully selected as it must not be too large due to real-time constraints, and at the same time, it should be adequately large to avoid high biases and variance [111]. In studies employing US-based signals, basic statistical features (e.g., mean and standard deviations) were extracted. Studies have also defined regions of interest in the USimage to extract different features [13]. When using NIRS-based sensors, the information recorded from the user is oxyhemoglobin and deoxyhemoglobin concentration changes. However, to extract meaningful information from these signals, studies extract a group of features comprising algebraic sums or differences on the raw data. Studies have also calculated some frequency domain signals, such as power spectral distribution and spectrograms of oxyhemoglobin and deoxyhemoglobin [76].

The properties of myography methods are wide. EMG-based methods have high temporal resolution. However, they are prone to sweat, fatigue and electro-magnetic noise in the environment. The MMG-based signals are not affected by sweat, have a higher signal-to-noise ratio, and are less sensitive to the variations in placement on the target muscles, but it is affected by the ambient acoustic and vibrational noise. US-based sensing provides a non-invasive method for tracking the movement of the deep-seated muscles, but current US devices are generally bulky and expensive, prohibiting its mainstream use in everyday life. NIRS-based sensing offers high spatial resolution while tracking user motion, but it is sensitive to muscle fatigue and optical noise. It also has a delayed response leading to a low temporal resolution. The fusion of myography methods brings different advantages to the interfacing framework. The EMG and MMG-based sensing methods acquire complementary information, as the signals acquired by the two sensing methods are generated at different times in the gesture execution cycle (EMG is generated as the cause of muscle motion and MMG is the effect of the muscle motion) [72]. Similarly, EMG and US signals are also generated at different times in the gesture execution cycle, with EMG as the cause of the motion and US-based data which records the effect of the motion. The fusion of US information with EMG also allows recording the motion information of deep-seated muscles which is not possible with just EMG. Fusion with NIRS gives insights regarding the assessment of neurovascular coupling during motion or grasp execution [77]. Fusion with accelerometers or IMUs provides a kinematic aspect of the user’s intention along with the dynamic aspects obtained by acquiring bio-signals from the user.

The right fusion method for the application at hand may be selected based on the aforementioned advantages and disadvantages. The most common fusion method is of EMG and accelerometers and EMG and IMUs, which can be attributed to commercially available bioamplifiers, such as Delsys Trigno (Delsys Inc., Natick, MA, USA) and Myoband (Thalmic Labs, Kitchener, ON, Canada). Therefore, the authors believe that the utilization of other fusion methods in research may be increased with an increasing number of commercially available sensors.

## 5. Conclusions

Following the PRISMA guidelines, we reviewed and analyzed the advantages and disadvantages of different myography methods and the potential of fusing them. The properties of myography methods are wide: Electromyography (EMG) data have high temporal resolution, mechanomyography (MMG) is robust to the skin–sensor interface, ultrasonography (US) allows the tracking of deep muscles in a non-invasive way without any crosstalk from adjacent muscles, while near-infra-red spectroscopy (NIRS) offers a high spatial resolution. In contrast, EMGs are non-stationary signals and prone to crosstalk with adjacent muscles and sweating and fatigue. MMG signals are prone to crosstalk between different muscle groups such as EMG and to interference due to ambient acoustic/vibrational noise. US-based interfaces are generally bulky and expensive and the probe requires frequent re-gelling for proper functioning and NIRS sensitive to muscle fatigue and optical noise and also has a delayed response to the motion of the user leading to a low temporal resolution.

All the studies included in this systematic review concluded that the fusion of two or more myography methods leads to a better performance in terms of decoding user intention. The study of myography fusion has been of continuous interest over the last decade. It was noticed that one of the most adopted methods’ for fusion is EMG-based signals with either accelerometers or inertial measurement units. The main focus has been on decoding the user intention for the upper limb. Furthermore, majority studies have focused on the discrete intention classification for both upper limbs and lower limbs, as opposed to continuous intention decoding. From this review, it can also be concluded that currently, the fusion of myography has mainly been explored for decoding discrete user intention (e.g., the classification of hand gestures and motions, walking terrains and patterns), while the continuous decoding of user intention remains relatively unexplored (e.g., continuous motion of human arm, force exertions). Therefore, future work should focus on the fusion of different myography methods to improve the user intention decoding during continuous tasks, such as decoding limb motion or decoding continuous user effort. For example, to decode continuous motions, EMG or MMG with accelerometers or IMU appears to be more promising, while in tasks that require both the classification and regression of user intention, a fusion of EMG and US can be employed. In future works, a fusion of MMG with US may be explored as well in such tasks. NIRS-based interfaces have difficulties in decoding forces exerted by the users while doing various tasks [100]; however, a combination of EMG, MMG, and NIRS may be used in recognizing the on-set of muscular fatigue [101]. One of the biggest challenges in comparing different fusion methods is currently the lack of standardized experimental protocol and assessment methods. Future studies may also focus on outlining such experiment protocols to allow the direct comparison of interfaces across different studies. We expect this to lead to a more intuitive and dexterous control of computer applications, as well as robotic and prosthetic devices.

## Figures and Tables

**Table 1:** Databases used for the literature survey and the search terms employed.

Databases	Search Term
Scopus, Pubmed, IEEE, Web of Science	(Skeletal Muscle OR Human Muscle) AND (Muscle Activity OR Electromyography OR Mechanomyography OR Sonomyography OR Myography) AND (Hybrid OR Multimodal OR Sensor Fusion OR Data Fusion) NOT (EKG OR EEG or Electrical Stimulation)

**Table 2:** Myography fusion methods explored in the studies included in the systematic review.

Fusion Method	Study	Properties of Fusion Methods
Fusion of EMG and MMG	Tkach and Hargrove [69] Fukuhara et al. [70] Tsuji et al. [71]	Provide complementary information regarding the intention [72]
Fusion of EMG and US	Botter et al. [73] Yang et al. [74]	Acquire information of both superficial and deep-seated muscles
Fusion of EMG and NIRS	Guo et al. [75] Guo et al. [39] Paleari et al. [76] Guo et al. [38]	Assess the same domain under different perspectives [77]
Fusion of EMG and Accelerometers	Fougner et al. [78] Roy et al. [79] Gijsberts and Caputo [80] Gijsberts et al. [81] Wu et al. [82] Joshi and Hahn [83] Gupta et al. [84] Wang et al. [85]	Dynamic and kinematic information of the user intentions
Fusion of EMG and IMU	Cannan and Hu [86] Wu et al. [87] Yang et al. [88] Fang et al. [89] Yu et al. [90] Zhou et al. [91]	Dynamic and kinematic (six or more degrees of freedom) information of the user intentions
Fusion of EMG and accelerometer with Optical Sensing	Yoshikawa et al. [92] Luan et al. [93]	Dynamic and kinematic information and complementary information to EMG data
Fusion of MMG and IMU	Woodward et al. [94] Woodward et al. [95] Ma et al. [96] Huo et al. [97]	Dynamic and kinematic (six or more degrees of freedom) information of the user intentions; combination is cheaper than using EMG [32]
Fusion of EMG, US and MMG	Chen et al. [98] Han et al. [99]	Provides complementary information regarding the intention and also acquires information of both, superficial and deep-seated muscles
Fusion of EMG, MMG and NIRS	Ding et al. [100] Sheng et al. [101]	Provides complementary information regarding the intention and assesses the same domain under different perspectives [77]

**Table 3:** Distribution of studies based on the topic included in each study. “X” denotes the topic included in that study.

Study	Myographies				External Sensors		
	EMG	MMG	US	NIRS	ACC	IMU	Optical
Fougner et al. [78]	X				X		
Cannan and Hu [86]	X					X	
Yoshikawa et al. [92]	X						X
Roy et al. [79]	X				X		
Tkach and Hargrove [69]	X	X					
Gijsberts and Caputo [80]	X				X		
Woodward et al. [94]		X				X	
Guo et al. [75]	X			X			
Chen et al. [98]	X	X	X				
Han et al. [99]	X	X	X				
Gijsberts et al. [81]	X				X		
Luan et al. [93]					X		X
Wu et al. [82]	X				X		
Guo et al. [39]	X			X			
Joshi and Hahn [83]	X				X		
Wu et al. [87]	X					X	
Woodward et al. [95]		X				X	
Ma et al. [96]		X				X	
Paleari et al. [76]	X			X			
Yang et al. [88]	X					X	
Guo et al. [38]	X			X			
Fukuhara et al. [70]	X	X					
Fang et al. [89]	X					X	
Gupta et al. [84]	X				X		
Wang et al. [85]	X				X		
Botter et al. [73]	X		X				
Ding et al. [100]	X	X		X			
Huo et al. [97]		X				X	
Yang et al. [74]	X		X				
Yu et al. [90]	X					X	
Zhou et al. [91]	X					X	
Sheng et al. [101]	X	X		X			
Tsuji et al. [71]	X	X					

## Data Availability

Not applicable.

## Abbreviations

The following abbreviations are used in this manuscript:

EMG	Electromyography
MMG	Mechanomyography
US	Ultrasonography
NIRS	Near-infrared wpectroscopy
IMU	Inertial measurement unit
MuMI	Muscle–machine interface
PRISMA	Preferred Reporting Items for Systematic Reviews and Meta-Analyses
